# Supplementary material for: Leptospirosis seroprevalence and exposure factors in three informal settlements of French Guiana: An opportunistic survey
Source: PLoS Negl Trop Dis. 2025 Nov 24;19(11):e0013764. doi: 10.1371/journal.pntd.0013764 (PMC12671760; doi:10.1371/journal.pntd.0013764)
Supplement: S1 Text — (PDF) [file pntd.0013764.s001.pdf]

S1 Text. Hantavirus Investigation in French Guiana 2022 – Questionnaire #1

To be completed for all participants who underwent sampling

Investigator
Name: .... First name: ....
Date of the interview (DD/MM/YYYY):
Investigation site: ☐ Boutillier, Rémire - Montjoly ☐ PK13, Macouria ☐ PK16, Macouria

Participant – Sociodemographic Characteristics
Date of birth (DD/MM/YYYY):
Country of birth: ....
Sex: ☐ F ☐ M ☐ Other
Residential area (map reference number): ....
Occupation: ....

Collective exposure factors – Only one response possible
What type of housing do you live in?
☐ Concrete/cement/cinderblock house
☐ Carbet (sheet metal, wood)
☐ Other, specify: ....

What is the main flooring in your home?
☐ Tiling
☐ Concrete
☐ Dirt
☐ Other, specify: ....
[If multiple floor types, indicate the flooring in the main living area]

Do you regularly go to the neighborhood water distribution point?
☐ Yes
☐ No
If no water point in the neighborhood, specify where water is collected: ....

Have you repeatedly observed rodents of this type* around your home since the beginning of the year?
☐ Yes
☐ No
If yes, were they more numerous at a certain time of the year, and when? ....

Have you repeatedly observed rodents or rodent droppings/urine inside your home since the beginning of the year?
☐ Yes
☐ No
If yes, specify the date of the most recent observation: ....

Are there waste materials stored around your home, whether or not in trash bins?
☐ Yes
☐ No

Individual exposure factors – Only one response possible
Do you regularly clean your house or neighborhood yourself?
☐ Yes
☐ No

Have you done cleaning work in poorly ventilated and infrequently used areas (storage rooms, sheds, attics) since the beginning of the year?
☐ Yes
☐ No

Do you usually perform agricultural or forest activities (e.g., wood cutting, grass cutting, soil work, planting, working in fields)?
☐ Yes
☐ No
If yes, which: ....

Do you use traps or poison against rodents at home?
☐ Yes
☐ No

Do you ever handle rodents (alive or dead)?
☐ Yes
☐ No

Symptoms compatible with recent Hantavirus infection – multiple answers possible
In the past 4 weeks, have you experienced any of the following symptoms? If yes, check all that apply:
☐ Fever > 38°C
☐ Chills
☐ Cough
☐ Severe headaches
☐ Shortness of breath
☐ Breathing difficulty
☐ Muscle aches
☐ Diarrhea
☐ Abdominal pain/nausea
☐ Vomiting

End of interview – Script

* using photographs of *Zygodontomys brevicauda* and *Oligoryzomys fulvescens* to assist participants with visual identification of the rodents vector of the hantavirus Maripa in French Guiana. The results of this question on specific rodents were not presented in this ancillary study.
