# Supplementary material for: Leptospirosis seroprevalence and exposure factors in three informal settlements of French Guiana: An opportunistic survey
Source: PLoS Negl Trop Dis. 2025 Nov 24;19(11):e0013764. doi: 10.1371/journal.pntd.0013764 (PMC12671760; doi:10.1371/journal.pntd.0013764)
Supplement: S2 Text — (PDF) [file pntd.0013764.s002.pdf]

S2 Text. Hantavirus Investigation in French Guiana 2022 – Questionnaire #2 (complementary questionnaire administered during the delivery of results) - To be completed for all participants who underwent sampling

Date of the additional interview (DD/MM/YYYY):
Investigation site: ☐ Boutillier, Rémire - Montjoly ☐ PK13, Macouria ☐ PK16, Macouria

Additional exposure factors – Only one response possible

Date of arrival in French Guiana (MM/YYYY)*:*
***Date of moving into the study area (MM/YYYY)*:** [*At least the year, ideally month and year]

Have you repeatedly observed rodents (all species) or signs of rodents (urine or droppings) in or around your home since the beginning of the year?
☐ Yes
☐ No

Do you use alternative sources of water for daily needs besides the neighborhood water distribution point?
☐ Yes
☐ No
If yes, which?
☐ Rainwater
☐ Well
☐ Creek
☐ Other, specify: ....

Is this water source covered?
☐ Yes
☐ No

Is this water source protected from flooding?
☐ Yes
☐ No

Do you frequently have wounds or damaged skin on your hands or feet?
☐ Yes
☐ No

Do you often walk barefoot or wear open shoes around your home?
☐ Yes
☐ No

If yes, do you also do so during heavy rain or when stagnant water is present?
☐ Yes
☐ No
