# Supplementary material for: Leptospirosis seroprevalence and exposure factors in three informal settlements of French Guiana: An opportunistic survey
Source: PLoS Negl Trop Dis. 2025 Nov 24;19(11):e0013764. doi: 10.1371/journal.pntd.0013764 (PMC12671760; doi:10.1371/journal.pntd.0013764)
Supplement: S1 Table — (PDF) [file pntd.0013764.s003.pdf]

S1 Table. List of 17 variables used for computing the cumulative exposure score

1	Walking barefoot or in open shoes during heavy rainfall or in stagnant water	**+1**
2	Frequent skin wounds on hands or feet	**+1**
3	Walking barefoot or in open shoes around household	**+1**
4	Using rain water collector as alternate source of water	**+1**
5	Using well as alternate source of water	**+1**
6	Using creek or running water as alternate source of water	**+1**
7	Using retention basin connected to a piped supply system	**+1**
8	Seeing rodent signs in or around household	**+1**
9	Having an at - risk occupation	**+1**
10	Practicing agricultural or forestry activities in contact with the ground	**+1**
11	Seeing waste near household	**+1**
12	Handling rodent (dead or alive)	**+1**
13	Household using of rodent traps or rodenticide	**+1**
14	Housing type different from house with concrete cement or cinderblock	**+1**
15	Primary flooring material of house hold different from tiling or concrete	**+1**
16	Not using a lid to cover the alternative water source	**+1**
17	Not using flood protection for the alternative water source	**+1**

Range of possible score: 0-17
