# Supplementary material for: Leptospirosis seroprevalence and exposure factors in three informal settlements of French Guiana: An opportunistic survey
Source: PLoS Negl Trop Dis. 2025 Nov 24;19(11):e0013764. doi: 10.1371/journal.pntd.0013764 (PMC12671760; doi:10.1371/journal.pntd.0013764)
Supplement: S1 Fig — (PDF) [file pntd.0013764.s004.pdf]

S1 Fig. Age distribution of participants in leptospirosis serological survey

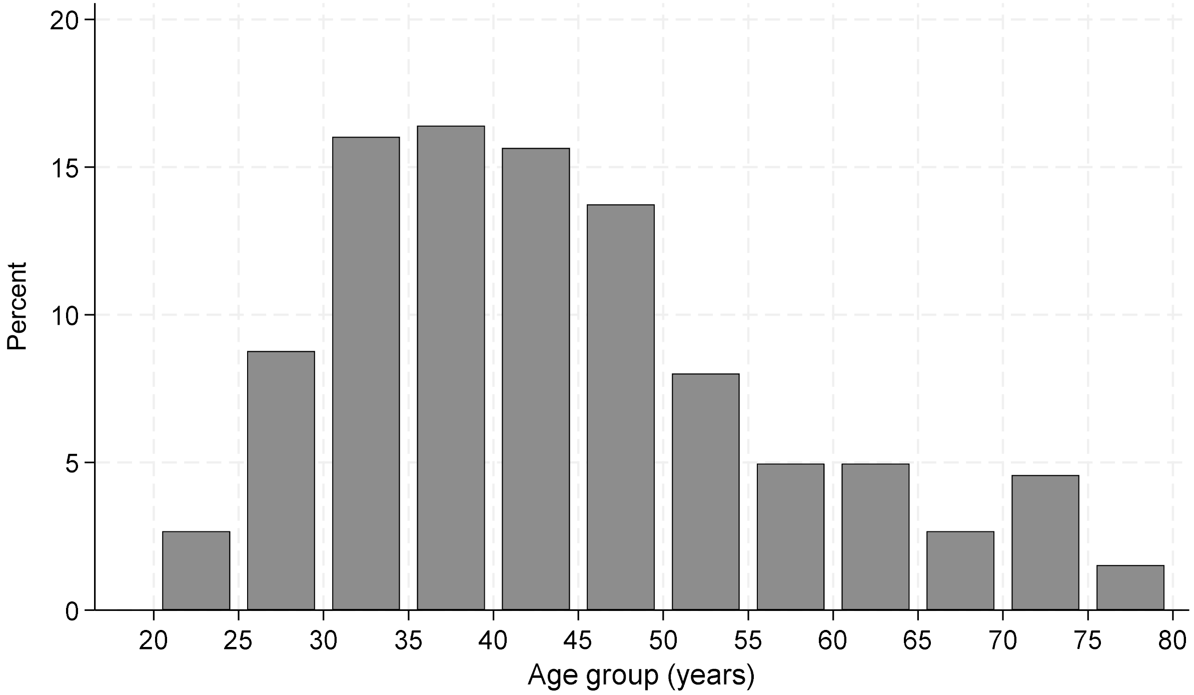

Participants aged <20 and >80 years were not represented in the figure
