# Supplementary material for: Leptospirosis seroprevalence and exposure factors in three informal settlements of French Guiana: An opportunistic survey
Source: PLoS Negl Trop Dis. 2025 Nov 24;19(11):e0013764. doi: 10.1371/journal.pntd.0013764 (PMC12671760; doi:10.1371/journal.pntd.0013764)
Supplement: S2 Fig — (PDF) [file pntd.0013764.s005.pdf]

S2 Fig. Distributions of the duration of living in French Guiana among study participants

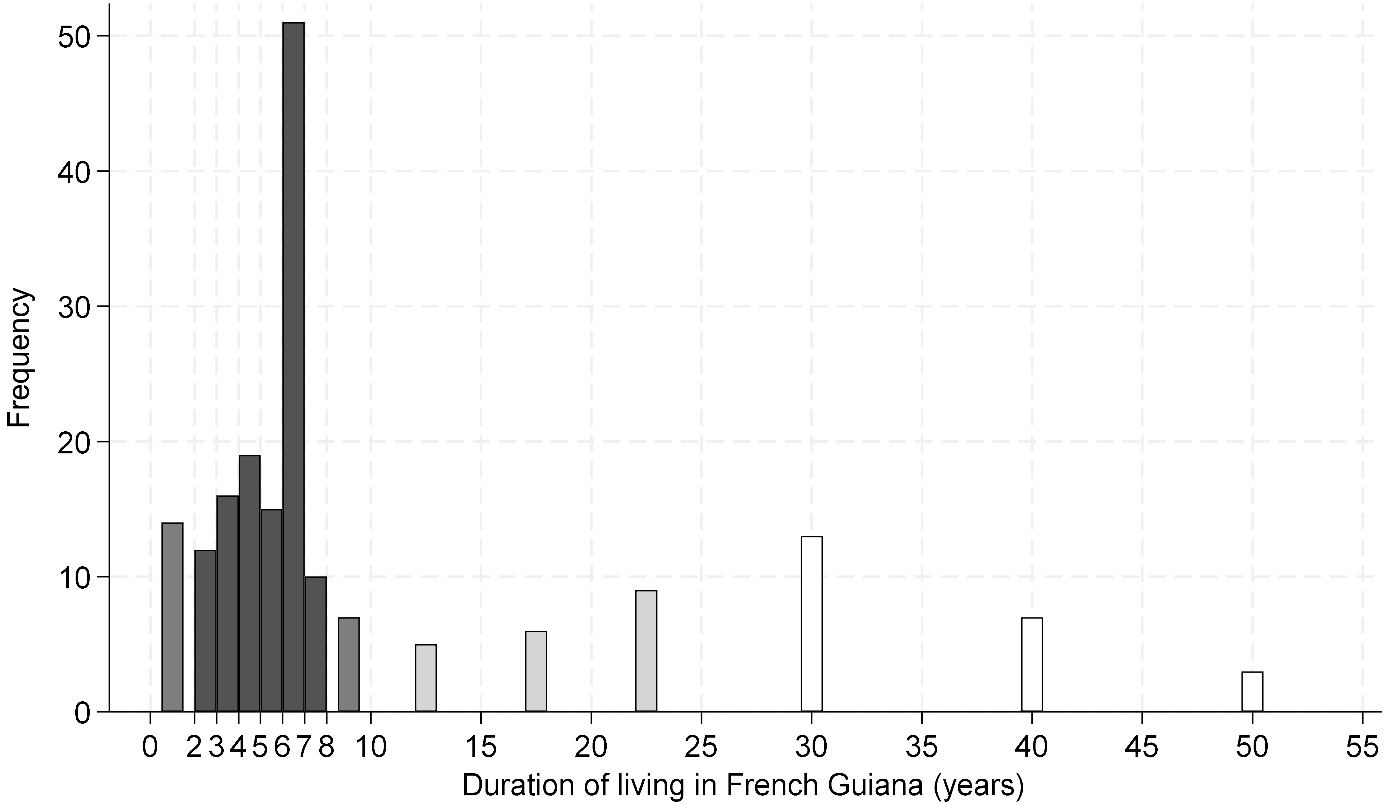

Data were aggregated to avoid categories with fewer than 5 participants as follows: dark grey 1 - year aggregate (2-8 years), medium grey 2 - year aggregate (0-2 and 8-10 years), light grey 5 year – aggregate (10-25 years) and white 10 - year aggregate (25-55 years).

Participants with a duration of residence in French Guiana longer than 55 years were not represented.
