# Supplementary material for: Leptospirosis seroprevalence and exposure factors in three informal settlements of French Guiana: An opportunistic survey
Source: PLoS Negl Trop Dis. 2025 Nov 24;19(11):e0013764. doi: 10.1371/journal.pntd.0013764 (PMC12671760; doi:10.1371/journal.pntd.0013764)
Supplement: S3 Fig — (PDF) [file pntd.0013764.s006.pdf]

S3 Fig. Distribution of the duration of residence in the study area among study participants

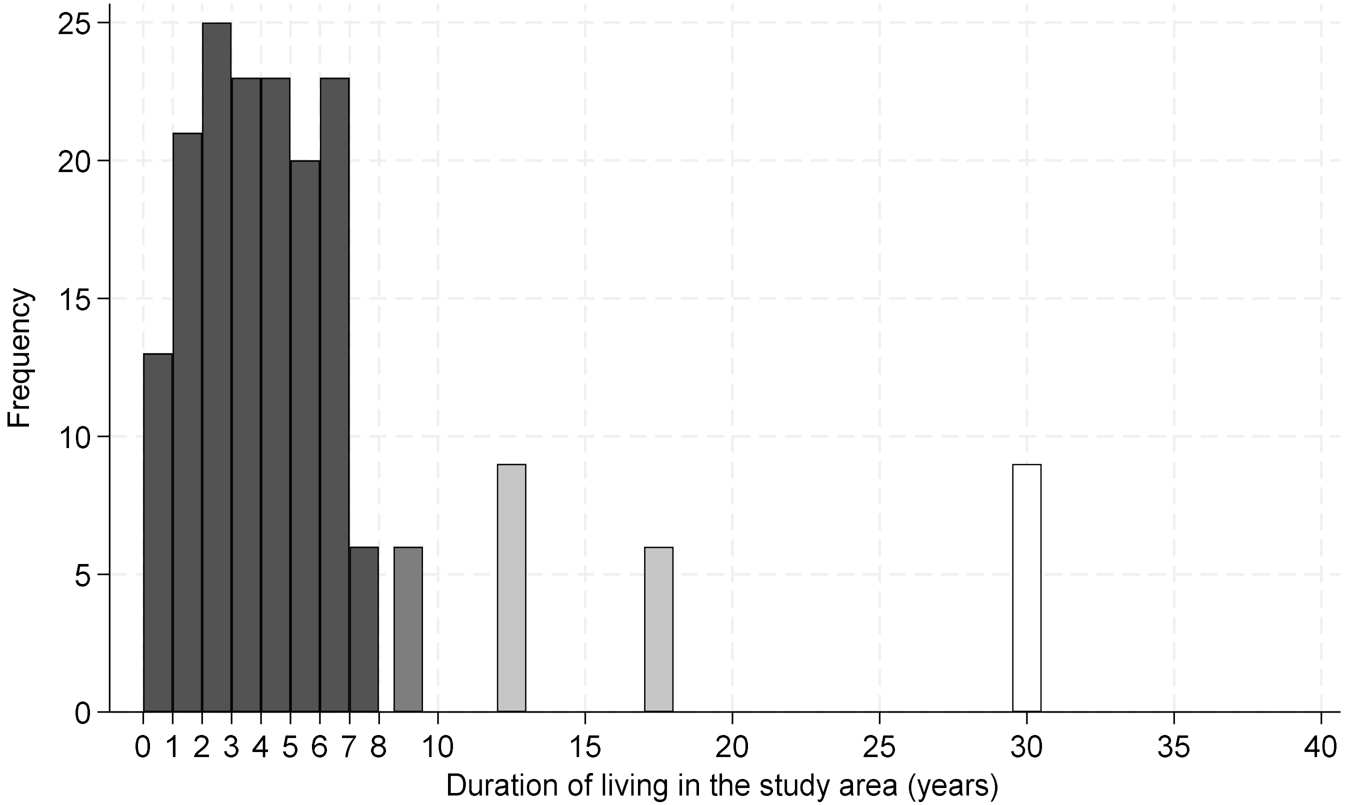

Data were aggregated to avoid categories with fewer than 5 participants as follows: dark grey 1 - year aggregate (0-8 years), medium grey 2 - year aggregate (8-10 years), light grey 5 year – aggregate (10-20 years) and white 20 - year aggregate (20-40 years).

Participants with a duration of residence in the study area longer than 40 years were not represented.
