# Supplementary material for: Leptospirosis seroprevalence and exposure factors in three informal settlements of French Guiana: An opportunistic survey
Source: PLoS Negl Trop Dis. 2025 Nov 24;19(11):e0013764. doi: 10.1371/journal.pntd.0013764 (PMC12671760; doi:10.1371/journal.pntd.0013764)
Supplement: S4 Fig — (PDF) [file pntd.0013764.s007.pdf]

S4 Fig. Water standpipe in close proximity to garbage deposits (*Photo credit: Margot Oberlis, Mobile Environmental Health Team, French Red Cross, Cayenne, French Guiana*)

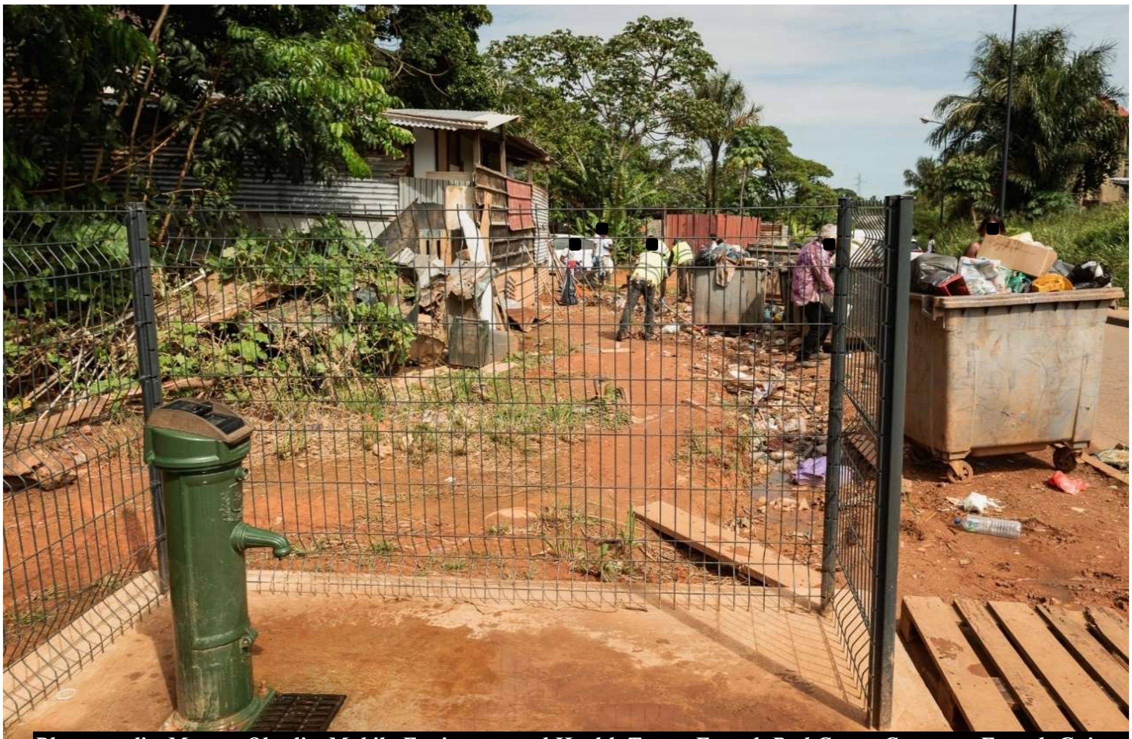

Photo credit: Margot Oberlis, Mobile Environmental Health Team, French Red Cross, Cayenne, French Guiana

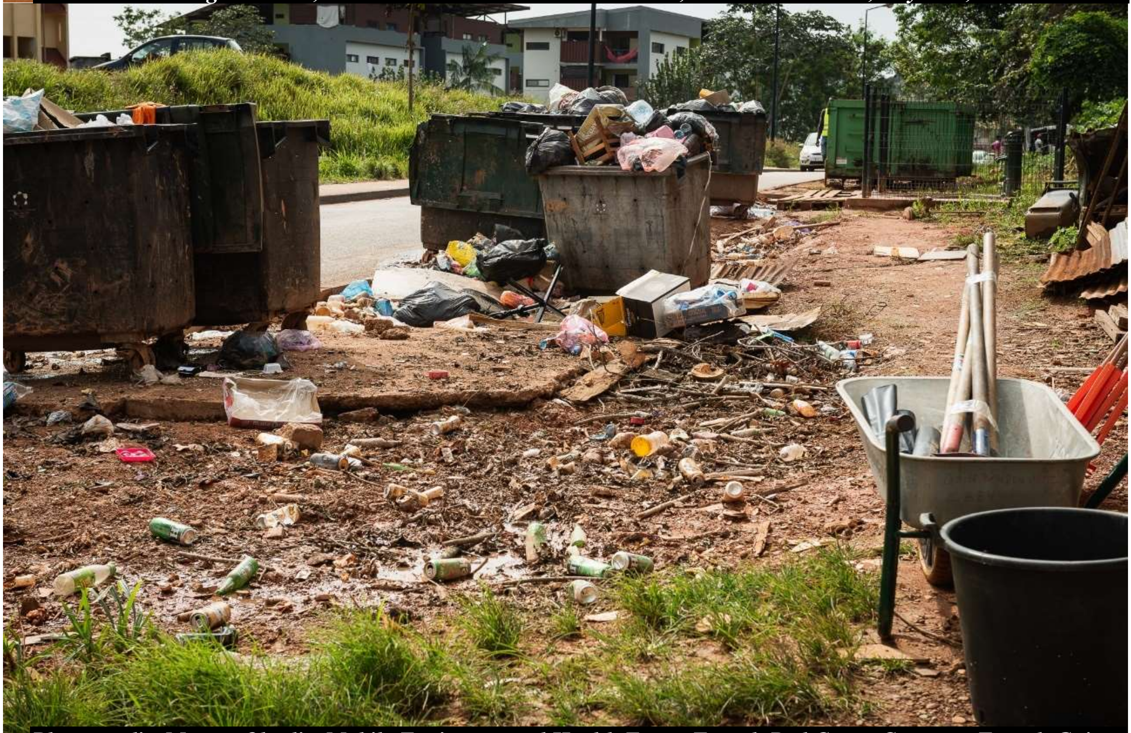

Photo credit: Margot Oberlis, Mobile Environmental Health Team, French Red Cross, Cayenne, French Guiana
