# Supplementary material for: Leptospirosis seroprevalence and exposure factors in three informal settlements of French Guiana: An opportunistic survey
Source: PLoS Negl Trop Dis. 2025 Nov 24;19(11):e0013764. doi: 10.1371/journal.pntd.0013764 (PMC12671760; doi:10.1371/journal.pntd.0013764)
Supplement: Attachment — Submitted filename: rebutal letter seroprev vf wo data avai.docx [file pntd.0013764.s009.docx]

Rebuttal letter

**Dear Editor,**

**Please find in bold and italics the responses to Reviewers' Comments.**

***Line numbering here refers to the clean version of the manuscript***

Reviewer #1: The objectives of the study are clearly articulated, the design is appropriate, population well described,

Reviewer #2: Abstract : In the abstract, it is difficult to understand the difference between the primary study and the ancillary study. It may not be necessary to specify this in the abstract.

***This part was removed from the abstract:***

***“A serological investigation was conducted in 2022 in three informal settlements in the area of Cayenne, the main city of French Guiana. Leptospirosis exposure factors were assessed in volunteers aged > 15 through standardized questionnaire. Leptospirosis seroprevalence was evaluated with Microscopic agglutination test (MAT) using 17 pathogenic Leptospira antigens with a reactivity threshold of 1:100.”***

Introduction : Line 92 and following : Here you assert the link between leptospirosis and poverty in French Guiana, but that is the research question of your work. I would be more cautious in assumptions here.

***The manuscript was modified as follows:***

***L 87***

***“Approximately one third of the affected individuals lacked health insurance, and a similar proportion lived in informal settlements—both figures higher than those observed in the general population. This overrepresentation of uninsured individuals and residents of informal settlements among leptospirosis cases suggests that the disease may disproportionately affect socially and economically vulnerable populations in French Guiana. Poor living conditions in urban informal settlements, particularly limited access to safe water and adequate sanitation, likely contribute to this increased risk. With few exceptions, residents living in informal settlements generally don’t have access to running water for drinking, food preparation, and other domestic uses. In these areas, inadequate wastewater and solid waste management leads to environmental contamination, including rudimentary disposal sites, insufficient garbage collection, and high rat populations. These factors provide a favorable setting for the emergence and transmission of leptospirosis. However, few data are available on the population living in these areas, apart from studies conducted by organizations involved in public health prevention, such as the Red Cross.[11,12]. Especially, no data exist in French Guiana on leptospirosis exposure among people living in informal settlements.”***

Line 129 : A map showing the location of these cases and the neighborhoods where the study was conducted, if possible with a representation of informal settlement areas, would be useful.

***The precise methodology used in this investigation around HPS cases as well as photographs of affected neighborhoods have already been reported. [12]***

***These photographs have been reported in the following open access article***

***Oberlis M, Guyot M, Le Turnier P, Carvalho L, Succo T, Rousset D, et al. The role of health mediation in investigation of Hantavirus cases among informal settlements inhabitants of Cayenne area, French Guiana, 2022–2023. Front Public Health. 2024;12. doi:10.3389/fpubh.2024.1364229***

***However, the authors are disposed to provide new photographs if this precision and reference is not deem sufficient by editors or reviewers.***

Line 153 : aide ?

***The term personal care aide was changed for Personal assistance***

Reviewer #3: 1. Line 132: Please provide an overview of the original survey’s sampling methods rather than referring only to a prior publication. It is not possible to assess the implications of the results without additional sampling details.
***The paragraph was modified as follows to provide more details on the methodology of participant recruitment:***

***L127***

***“******In 2022, an unusual number of cases of Hantavirus-associated pulmonary syndrome (HPS) was reported to health authorities of French Guiana.11 Four cases were diagnosed over a 7-month period while only 7 cases had occurred during the 2008 – 2021 period.12. This was considered as a possible severe emergence of the disease and led to conduct investigations around cases. After determining the place of living and probable place of contamination, three areas adjacent to Cayenne were selected for further on-site investigations. These sites were Boutilier in the municipality of Remire-Montjoly and an area named PK13 for kilometric point 13 and PK 16 for kilometric point 16 two sites in the municipality of Macouria. Multidisciplinary teams composed of health mediators, physicians specialized in infectious and tropical diseases and epidemiology and nurses were set up. The precise methodology used in this investigation around HPS cases has already been reported. 10 Awareness-raising sessions were conducted prior to the serological survey by health mediators and inhabitants were asked to participate during outreach visits. The inclusion criteria were age over 15 and residing in the study area (without duration limit). Participants were excluded if they declined participation in any ancillary study beyond the Hantavirus survey. The survey took place from 28th of November 2022 to 16th of December 2022. After providing information and obtaining written consent from participants, a questionnaire was administered. An information on the risk of rodent-transmitted diseases and protective measures as well as a hygiene kit were provided after performing the survey for each participant. Finally, several weeks after the initial survey, a campaign of results delivery was organized. Participants were approached individually by awareness-raising sessions but also through collective information campaigns at each of the three sites investigated. The Hantavirus serology results were communicated individually from 23rd of January to 27th of January.”***

2. Line 129: Clarify the number of informal settlements sampled. Based on the text (“Three areas near Cayenne were selected for on-site investigations: Boutilier [Remire-Montjoly] and PK13 and PK16 [Macouria]”), it appears that three areas within two informal settlements were included—please confirm.

***Boutiler , PK13 and PK16 were all three investigated. PK 13 and PK16 are informal settlements located in Macouria. However, they are distant from several kilometers and were considered as two different sites.***

3. Please summarize, in brief, the variables included in the cumulative exposure score to provide a general understanding of what factors were considered.

**We added to the methods section a short list of grouped factors that were considered.**

***L 194***

***“******Thus, a cumulative score was computed among the subgroup of participants in whom the complementary questionnaire was administered.*** ***The cumulative score was based on the following exposure factors: exposure to water and soil, water sources and management, rodent exposure, occupational and outdoor activities, housing and environmental conditions. The 17 variables used for computing the cumulative exposure score are given in supplementary data (see supplementary data S3).*** “

4. Clarify the languages used for survey administration (e.g., French only, or also Haitian Creole or others).

***This was added in the methods section***

***L145***

***“******French and Haitian Creole were the main languages used for survey administration. Guyanese Creole, English-speaking Guyanese Creole, Portuguese, Spanish and English were also used to a lesser extent***”.

5. Line 159: The following statement needs clarification: “It was initially anticipated to use a threshold of 1:50 for positivity threshold. However, many sera were contaminated and the MAT results were too complicated to interpret with this threshold.”
Please elaborate on how contamination influenced interpretability and the rationale for raising the threshold to 1:100.

***We thank the reviewer for this comment. The initial study protocol planned to use a positivity threshold of 1:50 for the Microscopic Agglutination Test (MAT). However, due to the poor quality of several serum samples—many of which were contaminated—it was difficult to obtain clear agglutination patterns at this dilution, making the interpretation of results unreliable. After review with the laboratory technicians, the MAT was therefore performed starting at a 1:100 dilution. This higher dilution helped reduce background interference and improve the readability of slides, allowing for more consistent and interpretable results.***

***The phrase was modified to gain in clarity as follows:***

***L 172***

***“******It was initially anticipated to use a positivity threshold of 1:50 for the Microscopic Agglutination Test (MAT). However, because many serum samples were contaminated, agglutination patterns were difficult to interpret at this dilution. The MAT was therefore performed starting at a 1:100 dilution, which reduced background interference and improved the readability of results.***

6. Line 106: Clarify the population to which the study objective refers in the statement:
“The primary objective of the study was therefore to assess the prevalence of prior Leptospira exposure in the population.”
Presumably this refers to residents of the three informal settlements, but this should be stated explicitly.

***Thank you for notifying this. Indeed, the statement was incomplete.***

***We reformulated as follows:***

***L 105***

***“The primary objective of the study was to assess the prevalence of prior Leptospira exposure in a population living in informal settlements near Cayenne using MAT serological analysis, and to investigate factors associated with seropositivity.”***

**Results**


-Does the analysis presented match the analysis plan?

-Are the results clearly and completely presented?

-Are the figures (Tables, Images) of sufficient quality for clarity?

Reviewer #1: Yes, Yes

Reviewer #2: It may be useful to specify that missing data were not considered in the calculation of odds ratios and p-values.

***We added this in the methods section to apply to the entire manuscript.***

***L 200***

***“All statistical tests were two-sided and a p-value of 0.05 was considered significant. Missing data were not considered in the calculation of odds ratios and p-values.”***

Reviewer #3: 1. Table 2: The reported N is 266, but the leptospirosis-specific questionnaire appears to have been administered to only 190 participants. Please clarify the denominator used for this table.

***The first questionnaire dedicated to hantavirus exposure was administered to the 266 participants with leptospirosis serosurvey. This first questionnaire comprised many variables that were also analyzed for leptospirosis exposure in this manuscript (see Supplementary 1).***

***We decided to remove “[..] participants in leptospirosis survey” throughout the manuscript to avoid confusion on whose participants we referred in the tables and figures? Besides it provided more concise table and figure titers.***

**Conclusions**


-Are the conclusions supported by the data presented?

-Are the limitations of analysis clearly described?

-Do the authors discuss how these data can be helpful to advance our understanding of the topic under study?

-Is public health relevance addressed?

Reviewer #1: Yes, Yes, Yes

Reviewer #2: Discussion:
Given the context of exposure widely shared by residents of these neighbourhoods, it would be interesting to supplement this study with seroepidemiological studies in nearby neighbourhoods with permanent housing.
***We thank the reviewer for these comments.***

***Seroepidemiological studies in nearby neighbourhoods would be very interesting indeed. We plan to conduct further analyzes on the general population of French Guiana using standardized and representative sampling design based on an existing biocollection called EPIARBO (see Bailly S, Hozé N, Bisser S, Zhu-Soubise A, Fritzell C, Fernandes-Pellerin S, Mbouangoro A, Rousset D, Djossou F, Cauchemez S, Flamand C. Transmission dynamics of Q fever in French Guiana: A population-based cross-sectional study. Lancet Reg Health Am. 2022 Oct 29;16:100385. doi: 10.1016/j.lana.2022.100385. PMID: 36777152; PMCID: PMC9903881).***

Line 293 : It might be useful to specify whether these seroprevalence studies were conducted in response to the occurrence of cases.

***L 319***

***“******These seroprevalences studies were conducted in response to occurrence of cases (ref) or because environment appeared favorable for leptospirosis transmission as done in French Guiana. (ref)”***

As the population is Haitian and may have been exposed in their country of origin and in transit countries (Suriname in particular), it would be interesting to discuss this and cite any references in these countries.

***This element was added to the discussion section as follows.***

***“Additionally, the time and place of acquisition of the previous exposure to leptospirosis cannot be ascertained since MAT remains positive for several years. [39] Unfortunately there is no data regarding the epidemiology of leptospirosis in Haiti to discuss the seroprevalence of the participants born in Haiti. Since 1965 the sole published study of leptospirosis cases from Haiti is a case series of three patients who needed air medical transport.[40] Most people born in Haiti who migrate to French Guiana travel through Brazil or Suriname. There are no published data on the epidemiology of leptospirosis in Suriname. In Brazil, numerous studies have investigated leptospirosis across various regions and contexts. However, the specific regions and duration of transit for each migrant participant were not assessed. For these reasons, further analyses on this aspect were not feasible.”***

Conclusion :
One cannot say that 7.5% is low; I would weigh it up.

***The remark is relevant so the conclusion was modified to .***

***L395***

***“This study confirmed a high rate of leptospirosis exposure factors but a rather limited seroprevalence among the surveyed population of three informal settlements of French Guiana.”***

Reviewer #3: The discussion should explicitly acknowledge or expand on the implication of the following limitations:

- Non-representative (convenience) sampling design

- High number of exclusions and/or missing data

- Limited capacity to generalize findings beyond the sampled communities

***As suggested by other reviewers we deeply reformulated the discussion to emphasize these points.***

***L362***

***“This study has several limitations,*** ***particularly the fact that many patients did not answer to the second questionnaire generating missing data and its non-random sampling design. All participants were recruited after being sensitized through outreach campaigns, either several days before sampling or on the same day just prior to participation. Consequently, we cannot exclude the possibility that this pre-intervention sensitization about a rodent-borne disease (HPS) may have biased some of the questionnaire responses. Nevertheless, more than 90% of sensitized inhabitants expressed their intention to participate, and nearly 80% eventually did so.[12] The attrition between sensitization and sampling was therefore limited, suggesting that inclusion bias among the sensitized population was minimal. The main limitation relates to representativeness and generalizability, since the outreach campaigns reached only about one-third of the estimated population across the three sites, despite the investigators’ extensive efforts. To better estimate the seroprevalence of leptospirosis and associated factors across French Guiana, larger and random sampling should be considered as performed for other zoonotic diseases.[37,38] Additionally, the time and place of acquisition of the previous exposure to leptospirosis cannot be ascertained since MAT remains positive for a long time. [39] Unfortunately there is no data regarding the epidemiology of leptospirosis in Haiti to discuss the seroprevalence of the participants born in Haiti. Since 1965 the sole published study of leptospirosis cases from Haiti is a case series of three patients who needed air medical transport.[40] Most people born in Haiti who migrate to French Guiana travel through Brazil or Suriname. There are no published data on the epidemiology of leptospirosis in Suriname. In Brazil, numerous studies have investigated leptospirosis across various regions and contexts. However, the specific regions and duration of transit for each migrant participant were not assessed. For these reasons, further analyses on this aspect were not feasible. Another limitation is that the study focused on peri domestic exposures, without extensively investigating extra-domestic activities - apart from occupational and agricultural activities but without detailing them. Many potentially relevant risk factors for leptospirosis-such as socioeconomic status, health literacy, prior disease knowledge, exposure to animals other than rodents, and local wastewater management-were scarcely or not assessed.***

***Finally, despite the methodological limitations of the study, the estimation of seroprevalence in understudied informal settlements and the identification of a local risk factor for exposure represent relevant findings for the local management of leptospirosis. Although their impact may be modest, these findings could support intervention campaigns around reported cases as well as broader awareness initiatives.”***

**Editorial and Data Presentation Modifications?**

Reviewer #1: The manuscript could benefit from some editing . A few examples are given below.
Material and Methods
Line 113-115: Health mediators helped the investigator and the participants when necessary during the on-field survey.
Please better specify the nature of ‘help’ provided. Did you mean to say: Health mediators assisted participants in filling out the questionnaire when necessary.

Discussion
Line 304-305: Because of a different titer threshold comparison was limited.
Consider: Since a different titer threshold was used, comparison may be questionable.

Line 342-343: Additionally, the time and place of acquisition of the previous exposure to leptospirosis cannot be ascertained since MAT remains positive for a long time.
Consider replacing ‘for a long time’ by ‘for several years’.

Reviewer #2: (No Response)

Reviewer #3: (No Response)

***We thank the reviewers for suggesting some grammatical modifications; We made the suggested modifications as well as some authors throughout the manuscript.***

**Summary and General Comments**


Reviewer #1: The manuscript is adequate. The subject does not importantly add to novelty in science, but is OK. It is well written. My comments on editing are included in the section above.

Reviewer #2: Thank you for the opportunity to review this manuscript. The article deals with an important topic in a geographical region where it has been little studied and provides original findings that improve our knowledge and are likely to have practical implications. In particular, the estimation of seroprevalence in these understudied informal settlements and the identification of walking barefoot on wet ground as a risk factor for exposure are important findings that can be used for prevention purposes. Overall, the article is well written, based on a clear and high-quality methodology, and fits well with the journal's themes.

Reviewer #3: 1. General Overview
The authors report on Leptospira seropositivity, assessed by microscopic agglutination test (MAT) using a 17-serogroup panel, among what appears to be a convenience sample of 190 individuals from three informal settlements surrounding Cayenne, the capital of French Guiana, in 2022. The original serological sampling and survey tools were designed for a hantavirus investigation following an apparent increase in cases in preceding years. The present study repurposes those samples and metadata, supplemented with a leptospirosis-specific questionnaire administered to the same participants. The principal finding is a leptospirosis seroprevalence of 7.5% (95% CI: 4.7–11.4) using a ≥1:100 MAT threshold—lower than anticipated given the multiple exposure risks associated with informal settlement living conditions.

2. Major Comments
Representativeness and Generalizability: While I commend the authors for deriving additional insights from existing samples, the non-systematic nature of participant selection raises major concerns about the representativeness of the data. Given the low observed seroprevalence, even small changes in case numbers could substantially alter the prevalence estimate.

Public Health Relevance: It remains unclear how the current findings can meaningfully inform public health policy or programming, given the convenience sampling design and limited external validity. The authors should clarify what practical insights—if any—can be reasonably drawn from these results.

***This is clearly a major limitation of the work. Indeed further studies are currently considered to use a set of biological samples that were collected through a standardized and representative sampling design.***

***The discussion was reformulated see above for the whole new paragraph on study limitations.***

***Regarding the practical insights from the study findings, the discussion was modified to:***

***L390***

***“Finally, despite the methodological limitations of the study, the estimation of seroprevalence in understudied informal settlements and the identification of a local risk factor for exposure represent relevant findings for the local management of leptospirosis. Although their impact may be modest, these findings could support intervention campaigns around reported cases as well as broader awareness initiatives.”***

3. Additional Comments

Title: Please specify that the study was conducted in three informal settlements. The current title implies coverage of all informal settlements nationally. A clearer version might read:
Leptospirosis Seroprevalence and Exposure Factors in Three Informal Settlements of French Guiana: An Opportunistic Survey.

***The title was modified as suggested to better describe at first sight what was done.***

Line 93: The following sentence requires elaboration to better delineate how leptospirosis disproportionately affects vulnerable populations: “A third of affected individuals lacked health insurance and a third lived in informal settlements indicating that leptospirosis disproportionately affects vulnerable populations.” I'm a little confused by the logic of this sentence. Can the authors explicitly clarify how A (A third of affected individuals lacked health insurance) and B ( third lived in informal settlements) lead to C (leptospirosis disproportionately affects vulnerable populations)

***We thank the reviewer for this comment. We added in the manuscript that these rates were higher than in general population and discussed further this point and reformulated.***

***L87***

***“Approximately one third of the affected individuals lacked health insurance, and a similar proportion lived in informal settlements—both figures higher than those observed in the general population. This overrepresentation of uninsured individuals and residents of informal settlements among leptospirosis cases suggests that the disease may disproportionately affect socially and economically vulnerable populations in French Guiana. Poor living conditions in urban informal settlements, particularly limited access to safe water and adequate sanitation, likely contribute to this increased risk.”***

Data availability: The current Data Availability statement appears internally inconsistent. Lines 394–395 state that data cannot be shared due to “ethical and legal restrictions,” yet lines 398–401 indicate that access to de-identified data may be granted upon reasonable request following approval from Santé Publique France and CNIL authorization. Further, the Data Availability section indicates that de-identified data can be accessed only after multiple institutional and CNIL approvals. Given that CNIL authorization governs identifiable data but does not restrict the sharing of fully anonymized datasets, it is unclear why the data cannot be made openly available in de-identified form. I encourage the authors to clarify whether these restrictions are institutional policy rather than legal requirement, and to consider providing an openly accessible anonymized dataset in alignment with PLOS NTD’s data-sharing standards.

***The data availability was indeed inconsistent.***

***The data availability was modified as follows:***

***“The datasets generated and/or analyzed during the current study were made accessible through an agreement with Santé publique France, and this agreement did not include provisions for making the data publicly available. Data processing and initial data collection were conducted under authorization No. 341194v42 granted by the French Data Protection Authority (Commission nationale de l’informatique et des libertés, CNIL) to Santé publique France. However, access to de-identified data may be granted upon reasonable request, after approval from Santé publique France and authorization from CNIL. Requests should be directed to the institutional data access committee via the Direction de la Recherche (DRCI) of Guyane University Hospital (email: drci.promotion@ch-cayenne.fr).”***
